# Endothelial nitric oxide synthase limits host immunity to control disseminated *Candida albicans* infections in mice

**DOI:** 10.1371/journal.pone.0223919

**Published:** 2019-10-31

**Authors:** Dhammika H. Navarathna, Michail S. Lionakis, David D. Roberts

**Affiliations:** 1 Laboratory of Pathology, Center for Cancer Research, National Cancer Institute, National Institutes of Health, Bethesda, Maryland, United States of America; 2 Fungal Pathogenesis Unit, Laboratory of Clinical Infectious Diseases, National Institute of Allergy and Infectious Diseases, National Institutes of Health, Bethesda, Maryland, United States of America; University of Michigan Health System, UNITED STATES

## Abstract

Three isoforms of nitric oxide synthase (NOS) occur in mammals. High levels of NO produced by NOS2/iNOS can protect against bacterial and parasitic infections, but the role of NOS in fungal innate immunity is less clear. Compared to wild type mice, *Nos3*^*-/-*^ mice showed significantly higher survival of candidemia caused by *Candida albicans* SC5314. NOS3/eNOS is expressed by endothelial cells in the kidney, and colonization of this organ was decreased during the sub-acute stage of disseminated candidiasis. *Nos3*^*-/-*^ mice more rapidly eliminated *Candida* from the renal cortex and exhibited more balanced local inflammatory reactions, with similar macrophage but less neutrophil infiltration than in infected wild type. Levels of the serum cytokines IL-9, IL-12, IL-17 and chemokines GM-CSF, MIP1α, and MIP1β were significantly elevated, and IL-15 was significantly lower in infected *Nos3*^*-/-*^ mice. Spleens of infected *Nos3*^*-/-*^ mice had significantly more Th2 and Th9 but not other CD4^+^ T cells compared with wild type. Inflammatory genes associated with leukocyte chemotaxis, IL-1 signaling, TLR signaling and Th1 and Th2 cell differentiation pathways were significantly overexpressed in infected *Nos3*^*-/-*^ kidneys, with *Nos2* being the most strongly induced. Conversely, the general NOS inhibitor N^G^-nitro-L-arginine methyl ester increased virulence in the mouse candidemia model, suggesting that iNOS contributes to the protective mechanism in infected *Nos3*^*-/-*^ mice. By moderating neutrophil infiltration, the absence of eNOS may reduce the collateral damage to kidney cortex, and Th-9 CD4^+^ cells may enhance clearance of the infection. These data suggest that selective eNOS inhibition could mitigate candidemia by a combination of systemic and local responses that promote a more effective host immune response.

## Introduction

Nitric oxide (NO) is an endogenous signaling molecule produced in mammals by three isoforms of NO synthase (NOS). Neuronal NOS (nNOS/*NOS1*) is synthesized by neuronal tissues and regulates the hypothalamo-neurohypophysical system [1]. Inducible NOS (iNOS/*NOS2*) expressed predominantly by macrophages and neutrophils in response to LPS and IFNγ signaling produces high (μM) concentrations of NO that mediate cytotoxic effects [2]. Endothelial NOS (eNOS *NOS3*) is constitutively expressed by endothelial cells, but its activity is highly regulated to yield nanomolar concentrations of NO that regulate vascular smooth muscle tone. Nanomolar concentrations of NO increase smooth muscle relaxation to increase blood flow and act on endothelial cells to increases vascular permeability and promote angiogenesis. Nanomolar levels of NO limit platelet activation to control hemostasis and have anti-inflammatory activity by inhibiting leucocyte adhesion [3]. NO produced by eNOS in renal endothelial cells [4] also plays important roles in preserving kidney function under hypertensive and diabetic stress conditions [5–7].

The higher concentrations of NO produced by iNOS interact with superoxide and other free radicals to product reactive nitrogen species (RNS) with potent anti-microbial activities against bacterial and parasitic infections [8–10]. For example, NO combined with peroxide protects against staphylococcus infection [11].

Although *C*. *albicans* and other pathogenic fungi can be killed by nitrosative stress and NO-releasing drugs [12,13], the role of endogenous NO production in host defense against fungal pathogens is less clear. A study of oral candidiasis in *Nos2*-null and WT mice found no differences in fungal clearance, host cytokine responses to the infection, or macrophage killing of *C*. *albicans in vitro* [14]. A similar lack of a significant *Nos2* null phenotype was reported following intranasal infection with the fungal pathogen *Coccidioides posadasii* [15]. However, resistance of *C*. *albicans* to host NO may result in part from expression of secreted NOS inhibitors and the NO-scavenging protein CaHYB1 by *C*. *albicans* [16,17] or from induction of arginase in host macrophages to prevent NO biosynthesis by depleting its substrate [18]. Selective sensitization of mice treated with an iNOS inhibitor to candidemia induced by a low virulence *C*. *albicans* MAP kinase mutant provides some evidence of a protective role of iNOS [19]. In contrast, *Nos2*-null mice were protected against systemic sporotrichosis caused by *Sporothrix schenckii* [20]. Resistance to *S*. *schenckii* infection in the *Nos2* nulls was associated with decreased IL-10 production, increased IFNγ, and increased lymphocyte proliferation. Splenocytes from infected *Nos2* null mice also exhibited less apoptosis, suggesting a maladaptive role of iNOS in immunity to this fungal pathogen.

In this study, we investigated whether the presence or absence of eNOS *in vivo* alters the susceptibility of mice to disseminated candidiasis. Remarkably, the absence of eNOS is associated with increased resistance towards disseminated candidiasis, which is associated with increased inflammatory cytokine expression and induction of Th_2_ and Th_9_ subsets. Although eNOS plays important roles in preserving kidney function under other stress conditions [5–7], our data demonstrate that lack of eNOS upregulates renal iNOS and limits renal pathology and colonization.

## Materials and methods

### Ethics statement

Experimental protocols, housing, and care of mice were conducted in an AAALAC approved facility in strict accordance with the recommendations in the Guide for the Care and Use of Laboratory Animals of the National Institutes of Health. The animal study protocol LP-022 was approved by the National Cancer Institute Animal Care and Use Committee. Humane endpoints were used to minimize suffering.

### Strains and growth conditions

For challenge of mice, *C*. *albicans* strain SC5314 [21] was grown overnight in 50 mL of Yeast Peptone Dextrose (YPD) medium at 30°C with aeration as previously described [22]. Cells were harvested by centrifugation at 3,000 g for 10 min, washed twice with 50 ml of sterile non-pyrogenic normal saline (Quality Biological Inc., MD), and resuspended in 10 ml of saline before quantification using a Petroff-Hausser counting chamber. The cell suspensions were adjusted to the final concentration for parenteral administration using non-pyrogenic sterile saline.

### Mouse infection with *C*. *albicans*

Inbred 8 to 12-week-old (20–25 g) wild type (WT) and *Nos3*^*-/-*^mice on a pure C57BL/6J background (The Jackson Laboratory) were bred in a NCI vivarium and used for all animal experiments. *Nos3*^*-/-*^mice were periodically back-crossed against WT to minimize phenotypic drift. SNP analysis of the *Nos3*^*-/-*^ mice confirmed a background purity of 98.9% [23]. Mice were randomly allocated to groups of five animals per cage and provided *ad libitum* access to filtered water and standard mouse chow. Each group of mice was inoculated intravenously in the lateral caudal tail vein using a 30 gauge needle with a volume of 0.1 ml of saline containing 5x10^5^ *C*. *albicans* cells in the first experiments and 10^5^ *C*. *albicans* cells in subsequent experiments where survival bleeding or time point euthanasia was required [22,24]. Clinical signs of illness in each mouse were evaluated three times daily, and mice displaying arched back posture, sunken eyes, ruffled hair or dehydration were euthanized immediately by CO_2_ inhalation and processed for complete necropsy and collection of tissues for histopathological examination. To examine virulence in the absence of eNOS, we used 10 mice per group. As controls we used WT and *Nos3*^*-/-*^ mice administered with 0.1 ml of non-pyrogenic sterile saline (n = 5 per group). To examine the role of the pan-NOS inhibitor N^G^-Nitro-L-arginine methyl ester (L-NAME) during disseminated candidiasis, two groups of mice with 15 mice per group were infected i.v. with 10^5^ *C*. *albicans* cells in 0.1 ml sterile non- pyrogenic saline. One group was provided drinking water containing 0.8 mg/ml L-NAME *ad libitum* [25]. Two groups of control mice with one group administered with 0.1 ml of non-pyrogenic sterile saline and one group provided with the same dose of L-NAME were used as controls for the infected mice.

Analysis of the initial cytokine response was conducted using WT and *Nos3*^*-/-*^groups of mice (n = 7 per group) infected with *C*. *albicans*. Retroorbital bleeding and tail clipping on day 1, 2 5 and 7 was done alternatively to initially screen immune response in candida infected mice to minimize the number of mice required.

To longitudinally monitor effects of *Nos3*^*-/-*^ on organ burden and host immune responses, mice infected with *C*. *albicans* were euthanized sequentially from 1 to 6 days post-inoculation (PI). A total of 18 mice from each strain were inoculated with SC5314, and 3 control mice received no fungal challenge. Three animals from each group were sacrificed daily from day 1 to day 6 PI for histopathology and cytokine assays. The 3 control animals, i.e., untreated and uninfected, administered with 0.1 ml of non-pyrogenic sterile saline were sacrificed, and the organs and serum were collected. The mean results for these 3 control animals were used as time zero values. Sera were stored at −80°C until analysis.

Flow cytometric analysis was used to quantify the inflammatory response in spleens, kidneys and brains from WT (n = 5) and *Nos3*^*-/-*^ (n = 5) mice at day 1, 2, 3, 4 and 6 PI with *C*. *albicans* as compared with uninfected control mice. Serum samples collected from these experiments were used to analyses additional cytokine response. We analyzed leukocyte infiltration in kidneys and brain tissues at 3 days PI intervals using at least 4 mice per group.

### Necropsy and histopathology

Immediately after euthanasia, macroscopic changes were recorded, and the brain, heart, lungs, liver, spleen, and right kidney were immersed in buffered 10% formalin, processed for paraffin embedding, sectioned at 5 μm, and stained with H&E. Grocott’s modification of Gomori’s methenamine silver (GMS) stain was used for detection of fungi *in situ* [26]. Inflammatory reactions were scored between ++++ for severe inflammation with PMN infiltration and + for localized inflammatory foci.

Histopathology images from sections of formalin-fixed and paraffin-embedded tissues stained with Gomori’s methenamine silver or H&E were obtained using a light microscope (Olympus BX51) fitted with a digital camera (Nikon DXM1200F) and ScanScope XT digital scanner (Aperio). Images were processed with Adobe Photoshop and Aperio ImageScope v11.1.2.760 (Aperio).

### Organ burden quantification

Three mice from each group were euthanized to examine inflammatory response and the cytokine study were used at days 2–6 PI to determine the fungal burden in their kidneys. After sterile isolation, kidneys were weighed and homogenized in 1.0 ml of nonpyrogenic sterile saline. 10 fold serial dilutions of 10^−2^, 10^−4^ and 10^−6^ in 0.1 ml of the homogenates were spread on triplicate on plates containing Nickerson’s medium, also known as BiGGY agar, a selective and differential medium for *C*. *albicans* [27]. After 48 h of incubation at 30°C, colony number, morphology, and color were recorded, and numbers of CFU per kidney were estimated. *C*. *albicans* appears as brown to black colonies with no pigment diffusion and no sheen [27].

### Determination of serum cytokines and chemokines

Serum was collected from sacrificed mice (unless otherwise noted) at various time points following infection with *C*. *albicans* in WT and *Nos3*^*-/-*^ mice. A Luminex bead array Milliplex MAP Kit (catalog no MPXMCYTO-70K, Millipore, Billerica, MA) was used to quantify IL-1a, IL-6, IL-10 IL-17, IL-12p40 TNF-β, MIP-1β and GMCSF. IL-9, IL-15, MIP-1α and MIP-1β Cytokines were analyzed in sera from an independent experiment, according to the manufacturer’s specifications.

### Mononuclear cell isolation

Single cell suspensions from brain and kidney were prepared after intracardiac perfusion of anaesthetized mice with 20 ml of normal saline to remove the circulating blood cells. Brain tissues were incubated with 1 ml of collagenase D (1 mg/ml; Roche) at 37°C for 30 min followed by mechanical disruption of the organs through a 100 μm filter. The homogenates were resuspended in 4 ml of 90% Percoll (GE Healthcare) in HBSS, and a gradient was prepared by overlaying 3 ml of 60% Percoll, 4 ml of 40% Percoll and 3 ml of HBSS respectively. The gradients were then centrifuged at 1,700 rpm for 18 min at 4°C, after which the band corresponding to mononuclear cells was isolated, and single cell suspensions were washed with HBSS and then resuspended in RPMI 1640 medium.

Kidney tissues were mechanically disrupted and passed through a 100 μm filter, after which the homogenates were resuspended in 8 ml of 40% Percoll, and gradients were prepared by overlaying the cell suspension on top of 70% Percoll (3 ml) in 15 ml tubes [28]. The gradients were centrifuged at 2,000 rpm for 30 min at 4°C, after which the band of mononuclear cells present at the 70%-40% interface was isolated and subsequently washed to make single cell suspensions. In all cases, the absolute number of mononuclear cells from each organ was determined prior to flow cytometry analysis. Splenocytes were prepared by mechanical disruption of spleens on 100 μm filters, after which the cells were treated with red blood cell lysis buffer (0.14 M NH_4_Cl and 0.017 M Tris-HCl, pH 7.2), and washed twice before staining. In all cases, the absolute number of mononuclear cells from each organ was determined prior to flow cytometry analysis.

### Flow cytometry

Mononuclear cells isolated from different organs were blocked with 3.3 μg/ml of anti-mouse CD16/CD32 (Fc block; BD Biosciences) in PBS containing 1% FBS for 20 min prior to antibody staining. The following antibodies were used to characterize leukocyte populations: CD45.2-FITC (BD Bioscience Clone 104), CD8-Pacific Blue (Invitrogen, Clone MH10), CD4 Qdot 605 (Invitrogen, Clone RM4–5), CD11b-PE-Cy7 (eBioscience, Clone M1/70), Gr1-APC (BD Bioscience, Clone RB6–8C5), CD11c-APC-Cy7 (Biolegend, Clone N418), Thy1.2 Alexa Fluor-700 (Biolegend, Clone 30/H12), NK1.1 PerCP Cy5.5 (BD Bioscience, Clone PK136), CD4-PeCy7 (Biolegend, GK1.5), and CD45.2-APCy7 (Biolegend, Clone 104). The primary antibody staining was performed for 30 min on ice in 100 μl of FACS buffer (PBS containing 1% FBS), the cells were analyzed using flow cytometry (Digital LSR II; BD), and the data were analyzed using FlowJo software (Tree Star, Inc).

For intracellular flow cytometry (IC flow) experiments, splenocytes were harvested from cohorts of WT and *Nos3*^*-/-*^ control and infected mice at 0–6 days PI. Single cell suspensions of splenocytes were stimulated with PMA/ionomycin (Sigma) for 2 h at 37°C followed by 2 h incubation in the presence of Golgi stop and Golgi plug (BD bioscience). Intracellular staining was performed to measure cytokine profiles of T cells from non-infected and infected mice. Briefly, splenocytes were treated with fixation buffer (eBioscience) for 30 min and then washed twice with permeabilization buffer. Cells were then stained with CD4-PE-cy5 (RM4–5), IL4-FITC (11B11), IL17-APC (eBio17B7), IL9-PE (RM9A4), and Foxp3-PB (MF-14), incubated at 4°C for 30 min, washed, resuspended in PBS+0.1% BSA+0.01% azide and analyzed using LSR II and FACS Diva software.

### Inflammatory gene expression

Inflammatory gene expression analysis was done in WT and *Nos3*^*-/-*^ mice at day 3 PI. Specific mRNA levels in spleen RNA were analyzed by NanoString methodology as previously reported [29,30] and conducted at the DNA sequencing core facility of NCI. Briefly, 100 ng of total RNA per kidney were hybridized to the target specific mouse inflammatory gene CodeSet at 65°C. The CodeSet contained probes against a panel of 179 genes encoding proteins involved in mouse inflammation and six internal reference genes and were used to analyze local inflammatory response in kidneys. The hybridized reactions were loaded onto the NanoString Prep station, which removes excess reporter, binds the reporter to the cartridge surface, and stretches the probes for scanning. Subsequently, the cartridges were loaded onto the NanoString Digital Analyzer and scanned. This method provides a quantitative analysis of gene expression [31–33]. Significantly upregulated gene list is shown in Table 1.

**Table 1:** NanoString analysis of inflammatory gene expression 3 days PI in spleens from infected *Nos3*^*-/-*^ mice. Results are presented as the fold-change relative to the respective gene expression in spleens from infected WT mice.

Gene	Fold change	P-value
***Nos2***	42.39	0.034
*Il12a*	25.14	0.025
*Ccr7*	4.68	0.008
*Tnf*	4.33	0.024
*Itgb2*	3.83	0.031
*Ltb*	3.36	0.004
*Il8ra*	3.27	0.024
*Ccl22*	3.18	0.008
*Cxcl9*	3.15	0.025
*Tlr7*	3.04	0.015
*Ccl19*	2.89	0.005
*Prkcb1*	2.86	0.026
*Cxcr4*	2.84	0.041
*Tlr6*	2.55	0.051
*Tlr1*	2.36	0.008
*Cd40*	**2.22**	0.009
*Tlr2*	2.20	0.046
*Ccr2*	2.04	0.014
*Tgfb3*	2.02	0.048
*Ccl8*	2.01	0.008
*C3ar1*	1.96	0.037
*Map2k6*	1.73	0.048
*Stat1*	1.63	0.051
*Il15*	1.57	0.045

The Excel-based method described by the manufacturer or the delta-delta Ct method was used to perform normalization compared to six internal controls and basic statistical analysis of the data. The normalized results are expressed as the relative mRNA level, and values for infected WT and *Nos3*^*-/-*^ spleens were averaged and shown as mean ± s.d. Statistical significance was calculated using Student’s *t* test with significance as p<0.05. Using Genego software in MetaCore [30], up- and down-regulated genes clusters were analyzed for significant pathways.

### Statistics

The probability of survival as a function of time was determined by the Kaplan-Meier method, and significance was determined by the log-rank (Mantel-Cox) test and Jehan-Breslow-Wilcoxon test using GraphPad Prism software. Serum cytokine expression patterns and flow cytometry data among all treatment groups at various time points were analyzed by two-way ANOVA with post Bonferroni comparison test. Three to 4 randomly selected mice from each group were euthanized at each time point for longitudinal comparisons. Data were analyzed for significant differences by comparing means of each triplicate reading at various time points assuming that the cytokine expression levels within each group of mice are normally distributed [34]. Absolute number of organ specific immune cell subtypes between WT and *Nos3*^*-/-*^were analyzed using Student’s t test.

## Results

### *Nos3* null mice are resistant to disseminated candidiasis

**Fig 1:**
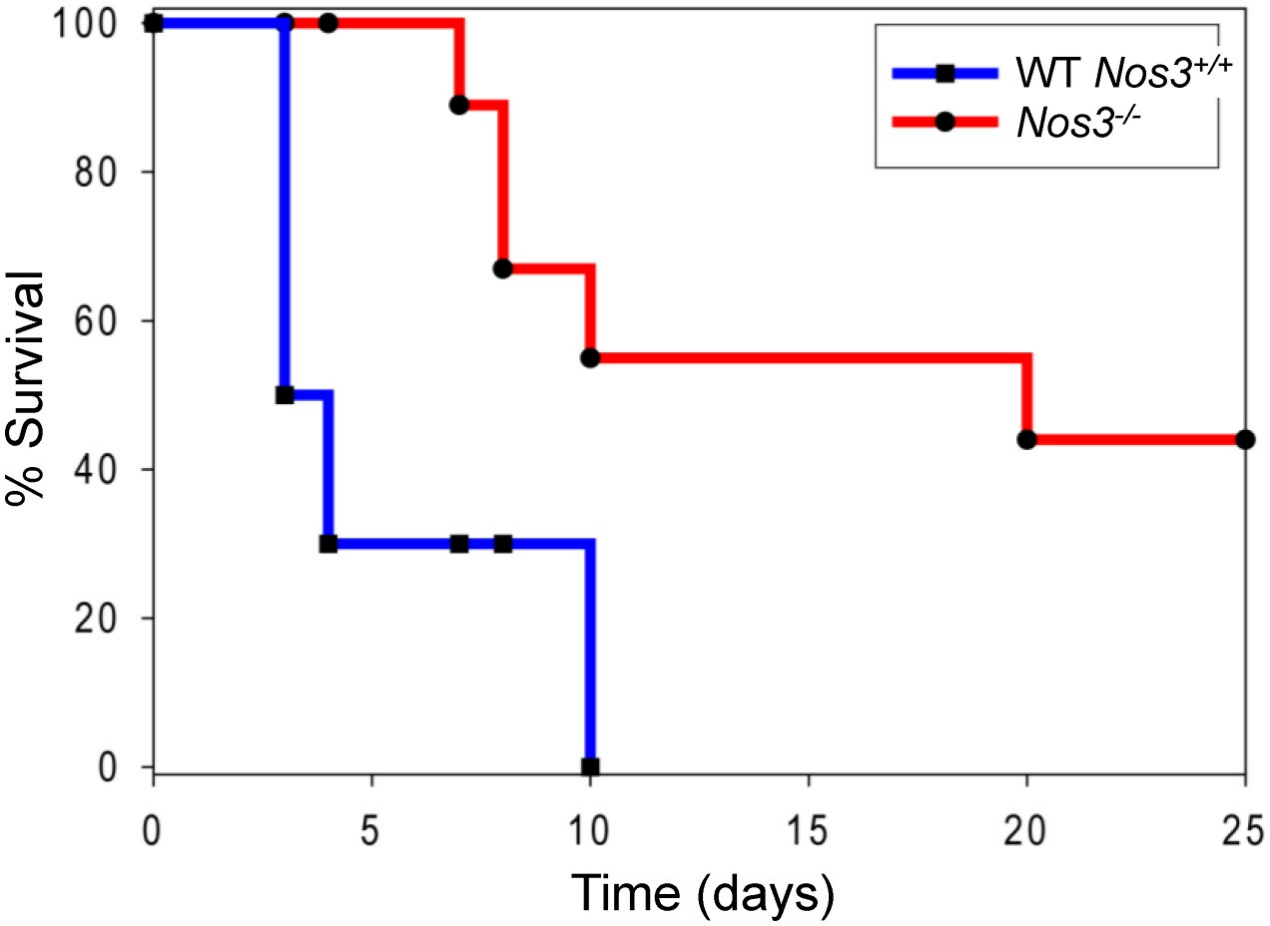
*Nos3* null mice are more resistant to disseminated *Candida* infection. Each group of ten 8–12 weeks old, 50% female and 50% male, mice were administered 5x10^5^ cells through the lateral tail vein, and survival was assessed using humane endpoints. Results were analyzed using log-rank (Mantel-Cox) test and Jehan-Breslow-Wilcoxon test. An uninfected control group of 5 mice was assessed daily after administering sterile saline i.v. and had no mortality.

A pilot study experiment using 8 mice per group showed that *Nos3* ^-/-^ mice infected with 5x10^5^ *C*. *albicans* had significantly higher survival compared with infected WT mice (p <0.02, hazard ratio estimates of 6.6 with 95% confidence interval of ratio 1.2–37.4). A repeat experiment using 10 mice per group confirmed that *Nos3*^*-/-*^ mice infected with 5x10^5^ *C*. *albicans* had significantly higher survival compared with infected WT mice (p <0.002, hazard ratio estimate of 7.2 with a 95% confidence interval of 2.1–25.1, Fig 1). WT mice inoculated with *C*. *albicans* died as early as 3 days post-infection (PI) and suffered 100% mortality by 10 days PI. Infected *Nos3*^*-/-*^ mice did not die until 7 days PI, and 50% survived at the end of the experiment on day 20 PI. Control mice administered with intravenous saline alone had no mortality.

### eNOS regulates circulating cytokine and chemokine responses

**Fig 2:**
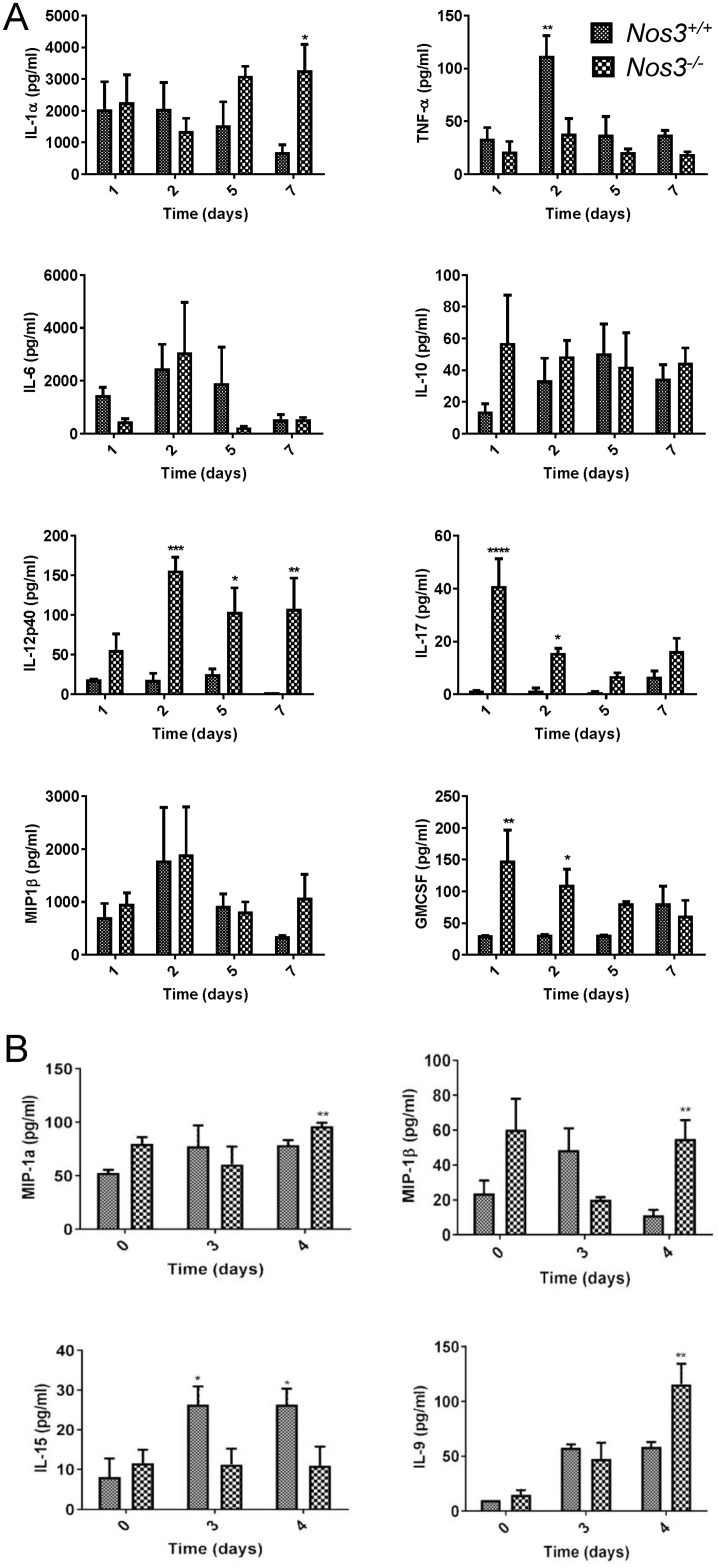
Effects of eNOS on systemic serum cytokines and chemokines during disseminated candidiasis. (A). Serum levels of the indicated proteins were assessed at 1 to 7 days PI for *Nos3* ^-/-^ and WT mice infected i.v. with *C*. *albicans*. WT (small checkered bars) or *Nos3* ^-/-^ (large checkered bars) data are presented as mean ± standard deviation for 7 mice per each group subjected to survival bleeding at each time point. (B) Serum levels of the indicated proteins were assessed in noninfected mice at day 0, and day 3, and 4 PI for WT and *Nos3* ^-/-^ mice infected iv with *C*. *albicans*. Mean values at time 0 were determined using sera from 3 control mice, and other data are mean ± standard deviation for 3 mice at each time point. (* = p<0.05; ** = p<0.01; *** = p<0.001, **** = p<0.0001).

To examine systemic responses underlying the increased protective responses in *Nos3*^*-/-*^ mice, we quantified several serum cytokines associated with innate immune responses up to 7 days PI. We used a sub-lethal dose of 10^5^ *C*. *albicans* to infect two groups of mice in order to perform survival bleeding while avoiding mortality reducing the number of evaluable mice. We observed that *Nos3*^*-/-*^ mice showed significantly increased IL-17, IL-12p40 and GM-CSF compared with WT infected mice (Fig 2A). Consistent with the improved survival in *Nos3*^*-/-*^ mice, IL-17 was significantly increased in *Nos3*^*-/-*^ mice at days 1,2 and 5 PI (p <0.0001, <0.05 and <0.05, respectively). Increases in serum IL-12p40 levels were also observed in *Nos3*^*-/-*^ mice at 2 and 7-days PI (p < 0.01 and <0.05 respectively). GM-CSF levels were significantly elevated in *Nos3*^*-/-*^ mice at day 1 PI (p<0.0001) compared to WT mice infected with the same dose of *C*. *albicans*. In addition, IL-1α decreased over time in infected WT mice but remained elevated in *Nos3*^*-/-*^ mice and showed a significant increase relative to WT at day 7 PI (p<0.05). In contrast, the transient significant elevation of TNF-β at 2 days PI in WT mice was absent in *Nos3*^*-/-*^ mice (p<0.01). IL-6 IL-10 and MIP1-β responses did not show significant differences between *Nos3*^*-/-*^ and WT mice (Fig 2A).

Serum was collected when mice were sacrificed at each timepoint for fungal burden, and systemic immune responses were assessed using a cytokine/chemokine panel to further define effects of eNOS expression on candida immunity (Fig 2B). Sera from *Nos3*^*-/-*^ mice contained significantly increased levels of the cytokine IL-9 (p<0.01), and the chemokines MIP-1α and MIP-1β (p<0.01) at day 4 PI. Conversely, WT mice expressing eNOS had significantly elevated IL-15 at day 3 and 4 PI that was absent in *Nos3*^*-/-*^ mice (p<0.05).

### Decreased kidney colonization and inflammation in *Nos3*^*-/-*^ mice

**Fig 3:**
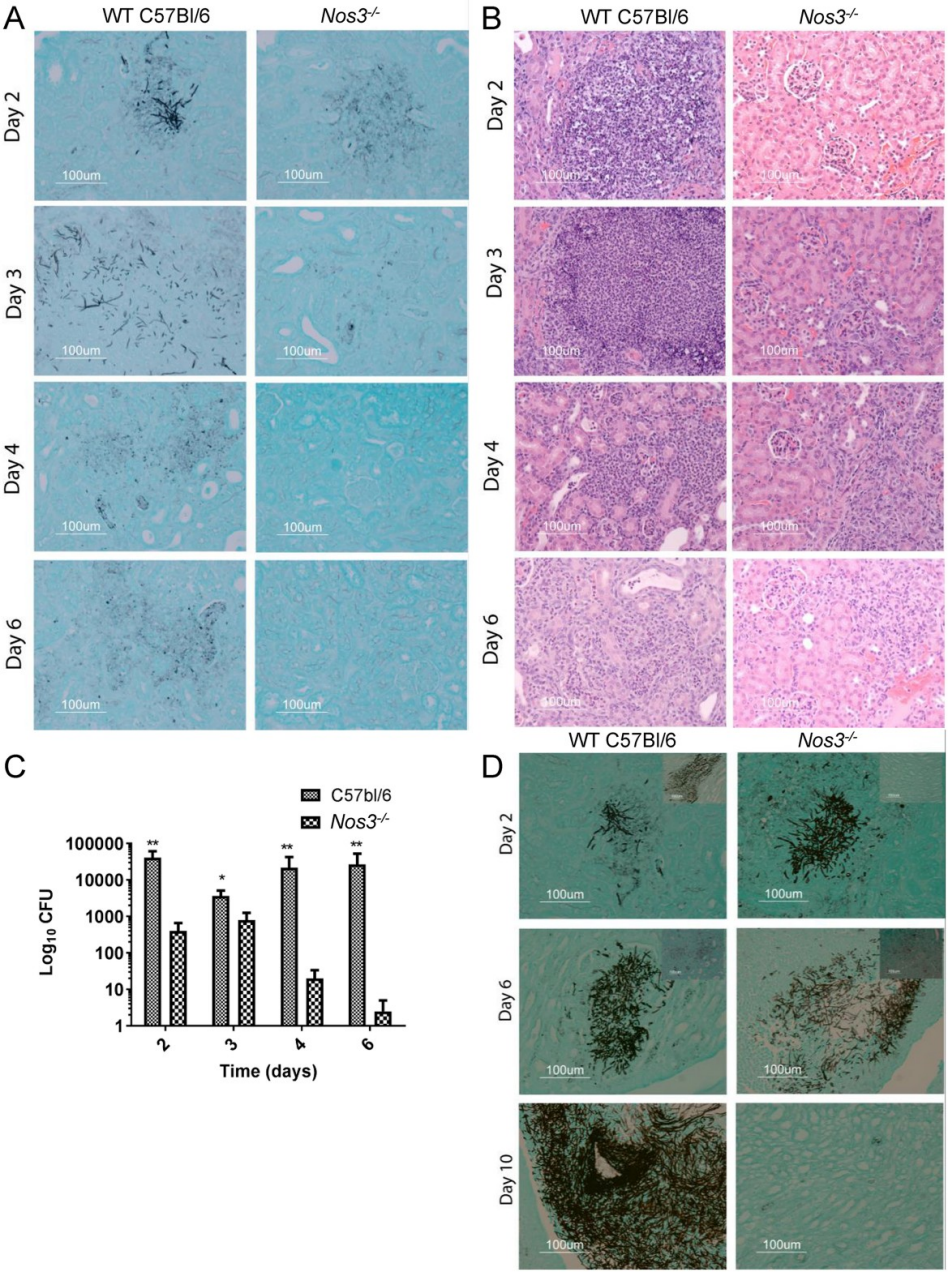
Kidneys of *Nos3* ^-/-^ mice infected with *C*. *albicans* show reduced fungal colonization and inflammatory responses. Representative sections of mouse kidneys stained using GMS to detect *C*. *albicans* colonization (black staining) in cortex (A) and medulla (D) and H&E stained cortex sections (B) to show inflammatory reactions in *Nos3*^*-/-*^ kidneys compared with the WT at days 2–6 PI. (C) Kidney fungal burdens were assessed in *Nos3*^*-/-*^ and WT mice infected with *C*. *albicans*. Small checkered bars represent mean CFU in left kidney homogenates from infected WT mice. CFU were assessed on BiGGY agar from six representative serial dilutions from each kidney, representing three mice per time point. Large checkered bars represent mean values for *Nos3*^*-/-*^ mice administered with *C*. *albicans*. * = p<0.05; ** = p<0.01.

Histological staining was performed to compare fungal colonization in kidneys from infected *Nos3*^*-/-*^ and WT mice. Kidney is the prime target organ for disseminated candidiasis. Fungal growth in kidneys was minimal in both groups of mice at day 1 PI, with no inflammatory reactions (S1 Fig). Mice euthanized at day 2 PI contained localized areas of mycelia scattered primarily in parenchyma of the kidney cortex (Fig 3A). At this time, we found no significant histological differences in colonization of WT and *Nos3*^*-/-*^ kidneys. However, as shown in representative H&E stained sections, WT mice kidneys infected with *C*. *albicans* displayed strong inflammatory reactions indicated by more polymorphonuclear cell infiltration, which were minimal in the corresponding *Nos3*^*-/-*^ kidney sections (Fig 3B).

By 3 days PI, the entire kidney parenchyma in infected WT mice showed severe pyelonephritis (Fig 3B) with invaded growing filaments, pseudohyphae, and yeast cells as shown in a representative GMS stained cortex section (Fig 3A), whereas infected *Nos3*^*-/-*^ mice showed comparatively less scattered colonization at 3 days PI (Fig 3A). Severe inflammatory reactions were observed in WT infected kidney cortex with polymorphonuclear leucocytes infiltration among necrotic patches, whereas the *Nos3*^*-/-*^ kidneys showed lower inflammatory reactions as shown in representative H&E stain sections (Fig 3B).

Greater differences were seen 4 days PI. *C*. *albicans*-infected WT mouse kidney cortex sections exhibited fungal cells with associated PMN cell infiltration, and *Nos3*^*-/-*^ mouse kidneys showed scattered rare fungal cells. However, we still observed inflammatory reaction in *Nos3*^*-/-*^ mice, suggesting effective fungal clearance in *Nos3*^*-/-*^ mouse kidneys (Fig 3A and 3B). In the kidney medulla of WT mice infected with *C*. *albicans*, we observed fungal colonization at day 2 and day 6 PI, whereas less *C*. *albicans* invaded the kidney medulla of *Nos3*^*-/-*^ mice at these times (Fig 3D).

At 6 days PI, the kidney cortex of WT mice showed sustained fungal colonization, while resolution was observed in kidney cortex tissues of infected *Nos3*^*-/-*^ mice (Fig 3A), and PMN became focally localized adjacent to the remaining organisms in the cortical region of WT kidneys (Fig 3B). *Nos3*^*-/-*^ mice infected with *C*. *albicans* at 6 days PI showed efficient cortical tissue clearing of fungal burden with milder inflammatory reaction (Fig 3A and 3B). At 10 days PI *C*. *albicans* heavily colonized the kidney medulla of WT mice but was cleared in the *Nos3*^*-/-*^ mice (Fig 3D).

Analysis of fungal CFU at the same time intervals of PI mirrored the histological findings and demonstrated that kidney colonization was largely cleared by day 6 PI in *Nos3*^*-/-*^ mice but was not cleared in WT mice. All the time points from day 2 to 6 PI kidneys of WT mice infected with *C*. *albicans* showed significantly higher CFU burden compared with the *Nos3*^*-/-*^ mouse kidneys at the corresponding time points (p< 0.01, 0.05 0.01 and 0.01, respectively, Fig 3C).

### Reduced kidney neutrophil infiltration and systemic immune response in infected *Nos3*^*-/-*^ mice

**Fig 4:**
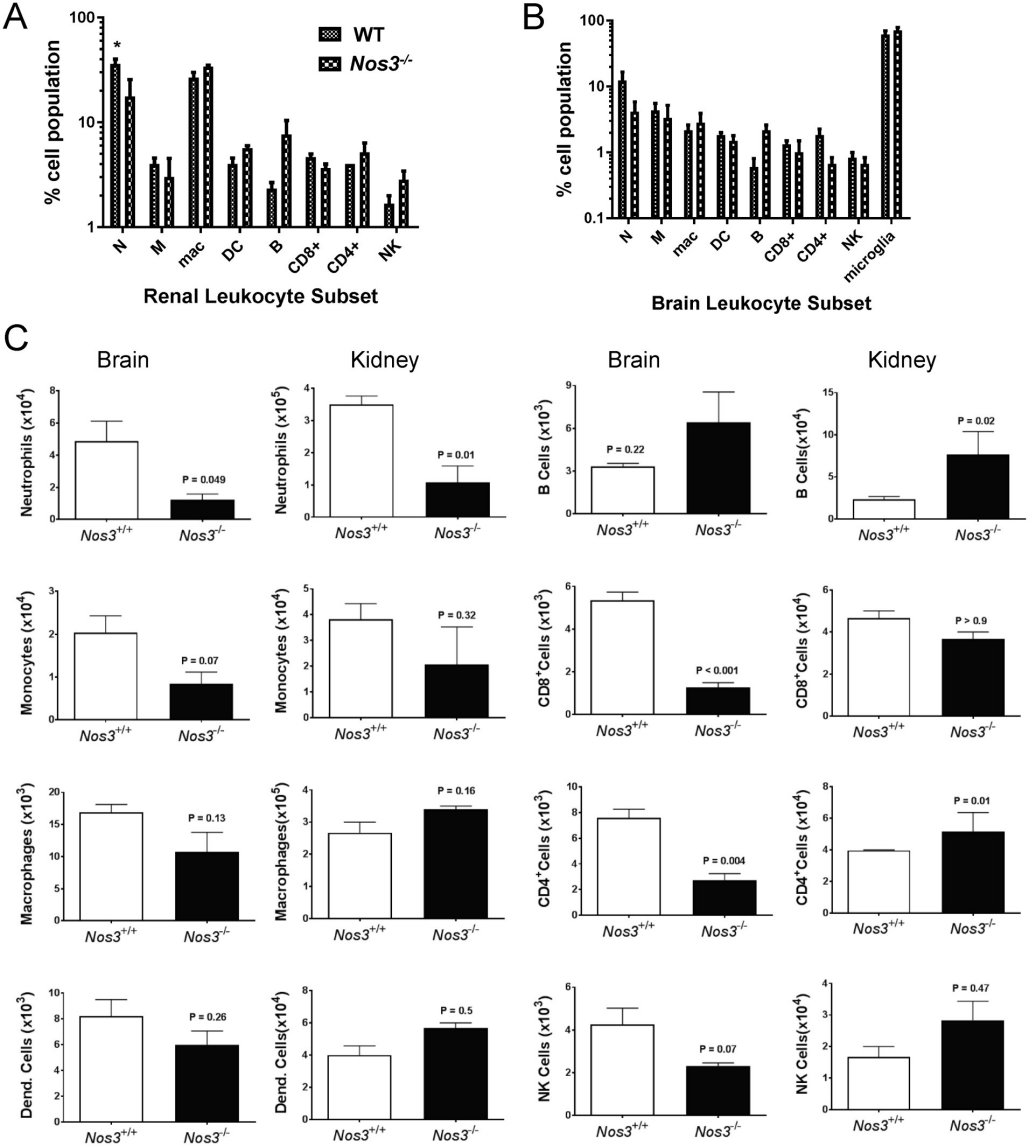
Flow cytometry analysis of mononuclear cell infiltration into infected kidneys and brains at day 3 PI in WT and *Nos3*^*-/-*^ mice. (A) Bar graph shows the percentages of infiltrated immune cells in kidney at day 3 PI (n = 5, * = p<0.05). (B) Bar graph shows the percentages of infiltrated immune cells at day 3 in brains from infected WT and *Nos3*^*-/-*^mice. (C). Bar graphs showing total infiltrating numbers of the indicated cell types in the infected brains and kidneys shown in A and B.

Based on our previous experience [35] and the histological estimation of inflammation in this study, we quantified local inflammatory response in infected kidneys and brain tissues using flow cytometry analysis of infiltrating leukocytes at 3 days PI (Fig 4). Percentages and absolute cell counts were analyzed as reported previously [36]. Examination of neutrophils, monocytes, macrophages, dendritic cells, CD8^+^ T cells, CD4^+^ T cells, and NK cells for their infiltration into kidneys showed that at day 3 PI, *Nos3*^*-/-*^ mice infected with *C*. *albicans* had significantly lower neutrophil accumulation on both a percentage basis and absolute cell counts (3-fold, p<0.05, and 4-fold, p<0.01, respectively) than did infected WT mouse kidneys (Fig 4A and 4C). Neutrophils were also decreased on an absolute basis in infected *Nos3*^*-/-*^ mouse brains (p = 0.049, Fig 4C). Although the increased percentages of B cells in infected *Nos3*^*-/-*^ mouse brain and kidney did not reach significance (Fig 4A and 4B), the number of B cells in *Nos3*^*-/-*^ kidneys was significantly higher (p = 0.02, Fig 4C). Absolute numbers of CD4^+^ cells were also higher in infected *Nos3*^*-/-*^ mouse kidneys p = 0.01), whereas CD4^+^ and CD8^+^ T cells were lower in *Nos3*^*-/-*^ mouse brains (p = 0.004 and <0.001, respectively), suggesting a diminished inflammatory response associated with *C*. *albicans* CNS infection in the absence of eNOS. All other immune cells lacked statistically significant differences.

### Loss of eNOS enhances systemic Th_2_ and Th_9_ responses to infection

**Fig 5:**
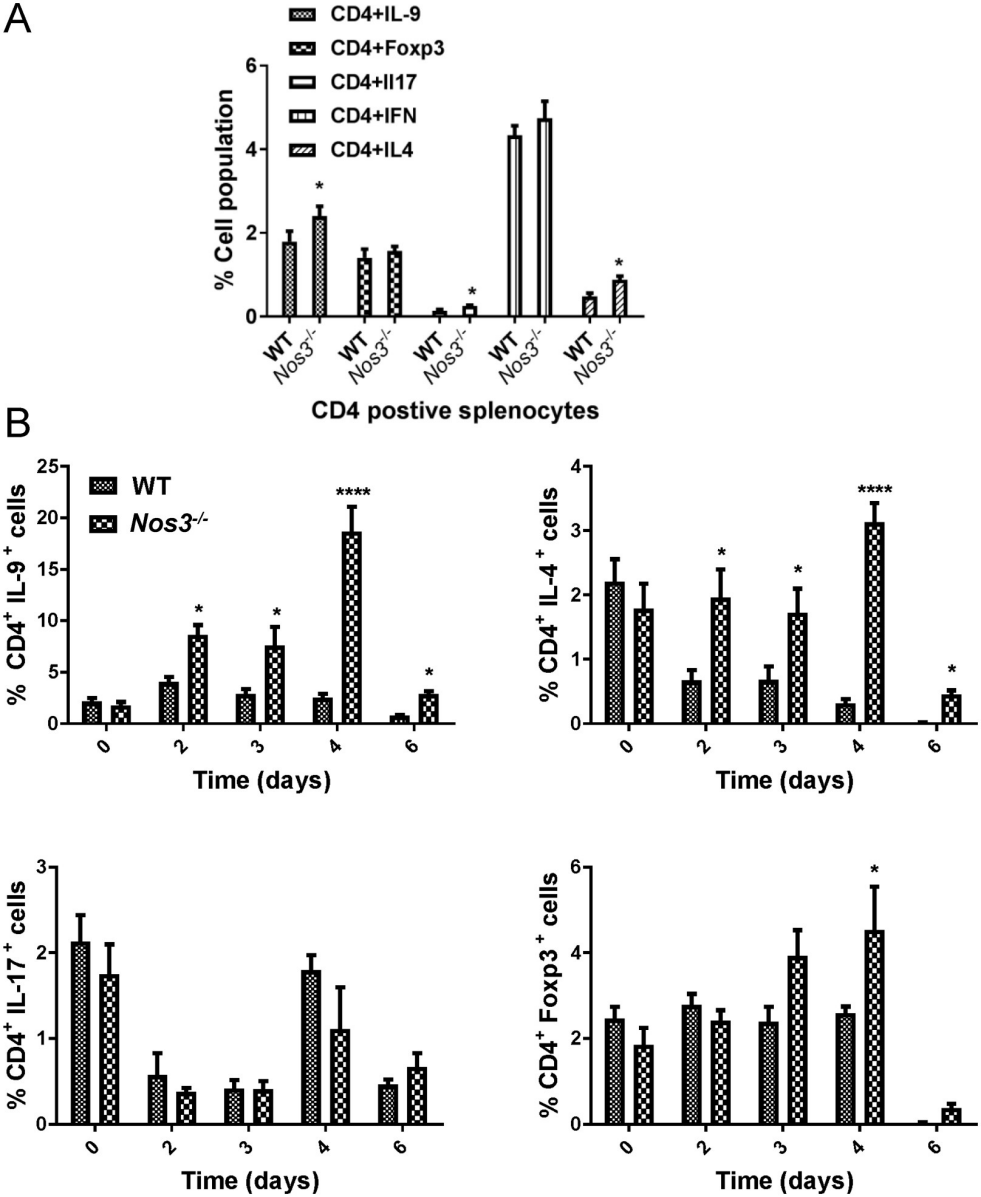
Th subset expansion in spleens of *Nos3*^*-/-*^ and WT mice infected with *C*. *albicans*. A. Cumulative bar graph of intracellular flow cytometry presenting the percentages of Th1 (IFNγ^+^), Th2 (IL-4^+^), Th9 (IL-9^+^) Th17 (IL-17^+^), and Treg (Foxp3^+^) CD4^+^ T cells in spleens from infected WT and *Nos3*^*-/-*^ mice at 3 days PI. Results from 5 mice per group were analyzed using two-way ANOVA with post Bonferroni comparison test. B. Follow up intracellular flow cytometry performed on splenocytes at days 2–6 PI and uninfected control groups (labeled as day 0 PI). The cumulative bar graphs based on flow cytometry analysis present the percentages of Th9 (IL-9^+^), Th2 (IL-4^+^), Th17 (IL-17^+^), and Treg (Foxp3^+^) CD4^+^ T cells in infected WT and *Nos3*^*-/-*^ mice. Results from 3 mice per group were analyzed using two-way ANOVA with post Bonferroni comparison test.

We analyzed CD4^+^ T cell subtypes in splenocytes at day 3 PI to assess the systemic immune response using intracellular flow cytometry. We observed that *Nos3* gene deletion alters host CD4^+^IL9^+^(Th_9_), CD4^+^IL17^+^(Th_17_), and CD4^+^IL4^+^(Th_2_) responses (Fig 5A). The significantly higher serum IL-17 and IL-9 levels in *Nos3*^*-/-*^ mice (Fig 2A and 2B) are consistent with induction of these CD4^+^ subsets in splenocytes from infected *Nos3*^*-/-*^ mice.

Based on the initial intracellular flow cytometry screen on 3 day PI, we conducted a comparative time point investigation of CD4 cell subset response of splenocytes of WT and *Nos3*^*-/-*^ mice as we reported in previous experiments [35]. Although the Th_1_ subset is considered to be protective against candidiasis [37], our data (Fig 5A) did not show differential Th1 response associated with *Nos3* genotype. Therefore, we further examined Th_2_, Th_9_, Th_17_ and T_reg_ T helper cells subsets in a detailed time point experiment (Fig 5B). Confirming our initial day 3 PI, intracellular flow, *Nos3*^*-/-*^ mice showed significantly higher Th_9_ subset (CD4^+^IL-9^+^) and Th2 subset (CD4^+^IL-4^+^) induction due to dissemination candidiasis at days 2–6 PI. *Nos3*^*-/-*^ mice showed a significant induction of T _reg_ (CD4^+^Foxp3^+^) cells at day 4 PI. No differential induction of the Th_17_ subset (CD4^+^IL-17^+^), which is considered to confer protective immunity to *C*. *albicans* [37], was observed between the two groups.

### Loss of *eNOS* upregulates iNOS in infected kidneys

To gain further insights into the mechanism by which eNOS alters local immune responses induced by *C*. *albicans* infection, we examined differential gene expression in infected kidneys at 3 days PI to be consistent with our previous reports [35,36]. Of the 193 inflammatory genes tested on the NanoString panel, 24 transcripts in kidney achieved significance comparing infected *Nos3*^*-/-*^ versus WT (>1.5-fold change with p <0.05, Table 1). iNOS mRNA was 52-fold upregulated in *Nos3*^*-/-*^ mice compared with WT (p>0.034). In addition, IL-12a was also remarkably upregulated in *Nos3*^*-/-*^ mice compared with WT (25-fold increase with p<0.025). Pathway-based gene expression analysis showed that eNOS-dependent genes in infected kidney tissues were associated with leukocyte chemotaxis (CCL19, CXCR4, CCR7, ITGB2 and alpha-L/beta-2 integrin), IL-1 signaling (iNOS, STAT1, TNF-alpha and MAP2K6), TLR signaling (TLR1, TLR2, TLR-6 and TLR-7), and immune response through IL-12 (CD40, IL-12 alpha chain and STAT1). Genes in these pathways were significantly overexpressed in infected *Nos3*^*-/-*^ mouse kidneys.

**Fig 6:**
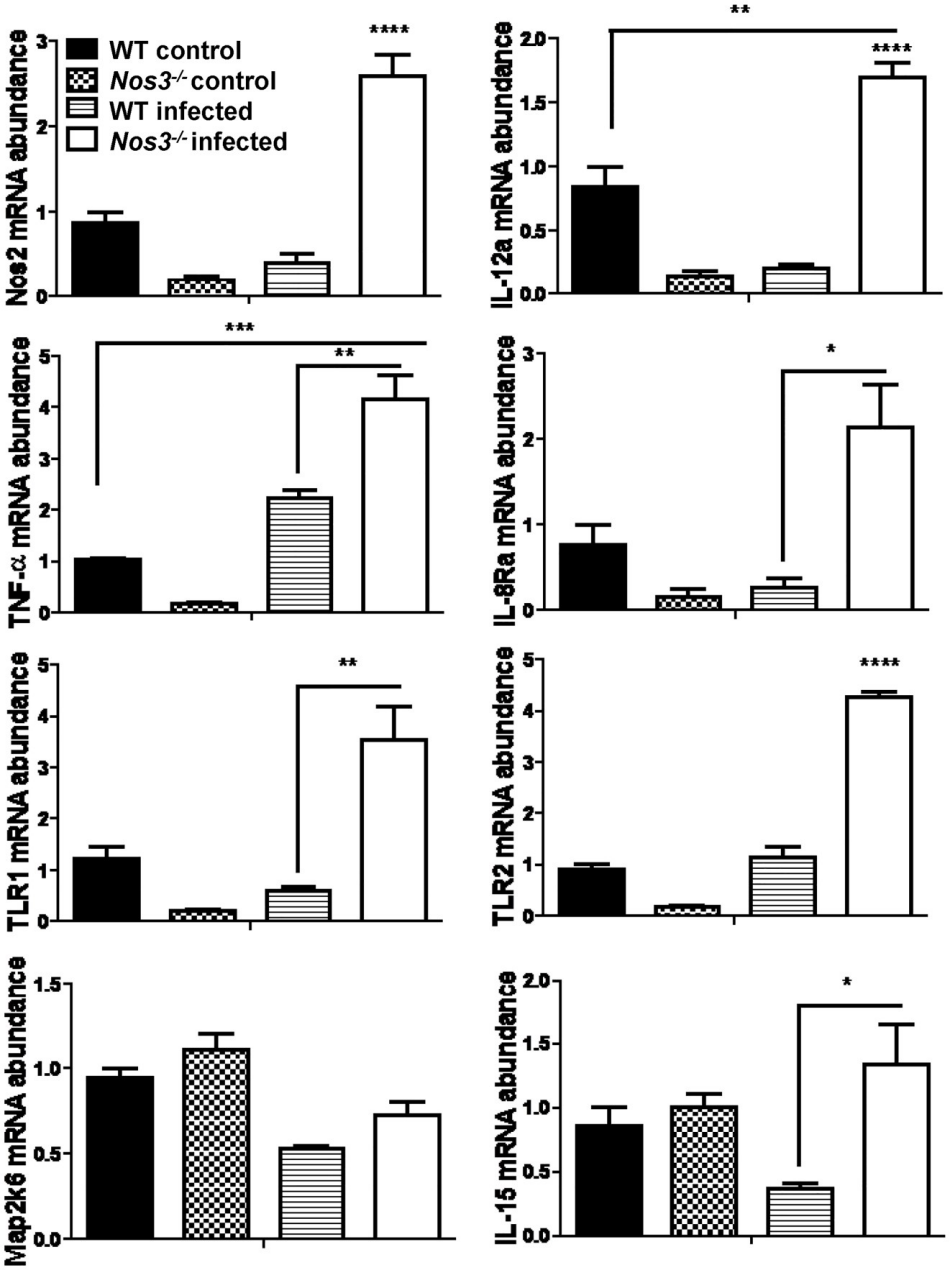
Effect of eNOS on local inflammatory gene expression induced in infected kidneys. mRNA abundance was determined by qPCR using cDNA synthesized from total RNA from kidneys to validate selected genes from the NanoString data in Table 1. Expression in uninfected WT and *Nos3*^*-/-*^ mice was compared with that in the infected WT and *Nos3*^*-/-*^ mice. Each group had at least three mice and confirmed that Nos2, Il-12a, TNF-a, IL-8Ra, TLR-1, TLR-2 and IL-15 expression in kidneys were significantly up-regulated in infected *Nos3*^*-/-*^ mice compared with the WT infected mice (* = p <0.05; ** = p <0.01; *** = p <0.001, **** = p <0.0001). Results from three mice per group were analyzed using two-way ANOVA with post Bonferroni comparison test.

In a separate experiment, real time PCR was used to validate expression patterns of selected genes identified in the NanoString analysis (Table 1) in *Nos3*^*-/-*^ vs WT infected and non-infected kidneys (Fig 6). In agreement with the NanoString data, mouse iNOS (*Nos2*) and interleukin 12a (*Il12a*, IL-12p35) were significantly up-regulated (p<0.0001). In contrast, we did not observe IL-12p40 (*Il12b*) as we observed in the systemic cytokine response. In addition, validation of expression for the selected genes *Tnf*, *Il8R*, *Tlr1*, *Tlr2* and *Il15* showed significantly higher mRNA level in infected *Nos3*^*-/-*^ mouse kidneys compared with WT (p<0.01, 0.05, 0.01, 0.0001 and 0.05, respectively). *Il15*, was validated to be significant, but *Map2k6* mRNA expression did not validate the NanoString analysis.

### Pan-NOS inhibition by L-NAME increases susceptibility to candidemia

**Fig 7:**
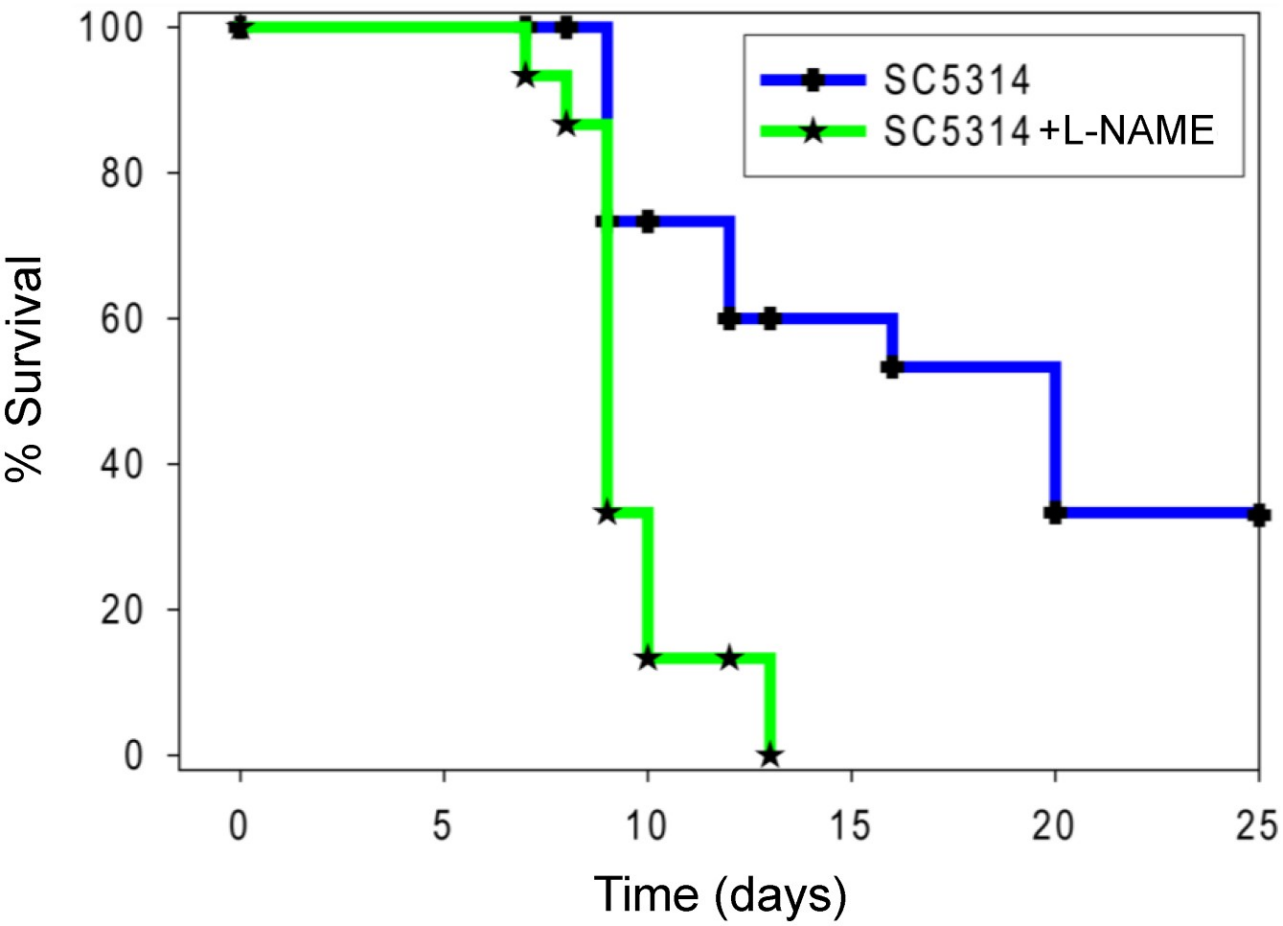
Pan-NOS inhibition by L-NAME increases mouse susceptibility to candidemia. The two treatment groups consisted of 15 8–12 weeks old mice and 50% female and 50% male. WT mice were administered 10^5^ cells through the lateral tail vein, and one group received L-NAME in the drinking water at 0.8 mg/ml concentration. Results were analyzed using log-rank (Mantel-Cox) test and Jehan-Breslow-Wilcoxon test. An uninfected control group of 5 mice was assessed daily after administering sterile saline i.v. and had 100% survival.

Although *Nos2*^*-/-*^ mice displayed no phenotype when challenged with oral candidiasis [14], the ability of an iNOS inhibitor to sensitize mice to a low virulence *C*. *albicans* mutant [19] and our gene expression data suggested that increased iNOS induction in colonized kidneys could contribute to the increased resistance of *Nos3*^*-/-*^ mice to candidemia and their clearance of the acute colonization. The correlation between impaired iNOS induction and impaired control of candidemia in mice lacking the C-type lectin CD23 provides precedent for such indirect function of iNOS [38]. To determine whether reducing overall NO production alters *C*. *albicans* pathogenesis in mice, mice were administered L-NAME p.o. during disseminated candidiasis. Confirming a net protective role for NO to control fungal pathogenesis, infected mice provided L-NAME in their water showed significantly higher mortality (p <0.01, hazard ratio estimate of 2.2 with 95% confidence interval of ratio 1.7–2.7 Fig 7). WT mice inoculated with *C*. *albicans* and receiving L-NAME died as early as 2 days post-infection (PI) and suffered 100% mortality by 13 days PI. Infected mice without L-NAME did not die until 4 days PI, and 33% survived at the end of the experiment on day 20 PI. Both groups of control mice administered with intravenous saline and p.o L-NAME alone had no mortality.

## Discussion

When challenged intravenously with *C*. *albicans*, *Nos3*^*-/-*^ mice in a C57Bl/6J background exhibited significantly reduced mortality compared with the WT. This is consistent with an almost complete clearance of viable *C*. *albicans* from its primary organ colonization in the *Nos3*^*-/-*^ kidney. In contrast, inhibiting all NO production using L-NAME in the same infection model resulted in increased mortality. Because the nanomolar concentrations of NO produced by eNOS are insufficient to generate cytocidal levels of RNS, we did not expect NO produced by eNOS to mediate a direct antifungal activity in disseminated candidemia. Rather, our data and existing precedent indicates that the levels of NO produced by eNOS in infected WT mice modulate multiple elements in the innate and adaptive immune responses that limit an effective defense.

Rapid clearance of fungal burden from the kidneys of *Nos3*^*-/-*^ mice may be mediated in part by the dramatic local induction of iNOS, which is well-documented to produce fungicidal levels of RNS [12,13]. Therefore, inhibition of protective RNS production by iNOS can account for the sensitizing effect of L-NAME treatment in our model, and this suggests that iNOS induction in the kidney is a major effector of the protective activity in absence of eNOS. This contrasts with a previous study that found bone marrow-derived macrophages from *Nos3*^*-/-*^ exhibit impaired induction of iNOS by lipopolysaccharide in vitro and that eNOS positively regulates iNOS induction in macrophages in a cGMP- and NFκB-dependent manner [39]. That data is consistent with the lower basal Nos2 mRNA we observed in uninfected mouse kidneys, but it also suggests that the increased Nos2 mRNA we see in infected kidneys did not result from a cell-intrinsic function of eNOS in infiltrating macrophages. Inflammatory signals can also induce iNOS expression via NFκB in renal proximal tubule epithelial cells [40,41]. These are the most abundant cells in the kidney cortex, and they also express eNOS [40,42]. Further studies are needed to determine which cell types in infected WT kidneys have altered eNOS activity and the mechanism by which this limits iNOS expression.

Immune cell infiltration in infected humans and WT mice can both protect from and aggravate kidney failure secondary to *C*. *albicans* colonization. The enhanced iNOS mRNA in infected kidneys of the *Nos3*^*-/-*^ mice cannot be attributed to increased macrophage infiltration. However, improved clearance of infected *Nos3*^*-/-*^ kidneys was associated with significantly lower neutrophil infiltration. Low neutrophil infiltration in kidneys of infected *Nos3*^*-/-*^ mice may provide a survival advantage based on prior evidence that *C*. *albicans*-induced neutrophil infiltration into brain and kidneys in WT mice causes fatal immune responses [36,43]. Lack of baseline organ infiltration of these immune cells is a limitation in this study. However, previously published data showed no basal difference in macrophage or CD3+ cell infiltration of kidneys in WT and *Nos3*^*-/-*^ mice [44]. Histology showed that colonization of kidneys in infected WT and *Nos3*^*-/-*^ mice diverged at day 3 PI, and the CFU assay showed improved clearance beginning at day 2 PI. However, histology suggested that more filamentous invasion in kidney cortex of WT mice as early as day 2. At this time, infected kidneys of *WT* mice exhibited excessive neutrophil infiltration as shown by histopathology and flow cytometry, which is consistent with the inflammatory responses observed in H&E stained sections of these kidneys. Early neutrophil recruitment (24–48 hrs. PI) has shown to play protective role in mouse disseminated candidiasis [45]. However, excessive neutrophil accumulation in tissue during the subacute phase of the infection is deleterious, and that immunopathology can result in kidney failure and mortality [46]. The brain also a target in candida dissemination [47], but the colonization in brain was insufficient to quantify. We focused instead on assessing infiltrating leukocytes, which revealed eNOS-dependent differences in neutrophil counts that paralleled those found in infected kidneys. Induction of iNOS was the strongest differential local inflammatory response observed in infected *Nos3*^*-/-*^ versus WT kidneys, and pan-NOS inhibition suggested that the RNS produced by iNOS mediate a protective response. Recent evidence suggests that this induction of iNOS in macrophages is mediated by CD23 [38]. Thus, in contrast to the previous bacterial renal infection models [48], *Nos3*^*-/-*^ mice displayed a decrease in neutrophil recruitment and acute upregulation of iNOS that increased survival together with protective systemic response.

In disseminated candidiasis, pathogen recognition receptors initiate complex signaling cascades to produce pro-inflammatory cytokines and chemokines that promote the leucocyte recruitment and differentiation, including induction of T helper cell responses [46]. Th1 and Th-17 subsets are reported to be protective, and Th2 to promote sustenance/persistence of candida [37]. Infected *Nos3*^*-/-*^ mice showed increased Th2 and Th9 induction compared with the WT. Defective Th2 and Th9 expression was reported in human patients with mucocutaneous candidiasis, suggesting their requirement for balanced immunity [49]. Although absolute numbers were not determined, the increased relative abundance of Th2 and Th9 CD4^+^ cells in spleens of infected *Nos3*^*-/-*^ mice suggest that these T helper subsets enhance protective immunity, and their role in human disseminated candidiasis merits further investigation.

Up-regulation of systemic IL-17 but not splenic Th-17 CD4^+^ cells was also associated with protection in the *Nos3*^*-/-*^ mice. IL-17 is a pro-inflammatory cytokine that is essential for host defense against candida [50]. IL-9 is a pro-inflammatory cytokine derived from Th-9 cells [49] that was consistently elevated based on our intracellular flow data and on day 4 PI in serum from infected *Nos3*^*-/-*^ mice, suggesting it contributes to protection against disseminated candidiasis. In contrast, granulocyte-macrophage colony-stimulating factor (GM-CSF) is a vital hematopoietic growth factor that induces functional activities of various circulating leukocytes [51]. Our 1 day PI serum cytokine data suggests that GM-CSF supports *Nos3*^*-/-*^ mice to mount a more protective innate immune response. In addition, the elevated chemokine MIP-1α/CCL3 at 4 days PI provides a chemotactic signal to promote recruitment of inflammatory cells and maintain a CD8^+^ effector immune response and B cells [52]. MIP-1b was also induced and is chemotactic for activated CD4^+^ lymphocytes [52]. *C*. *albicans* hyphae induce less MIP-1α and MIP-1β compared to *C*. *albicans* yeast [53], and this difference is consistent with the decreased filaments we found in kidneys of infected *Nos3*^*-/-*^ mice.

*Nos3*^*-/-*^ mice had significantly lower kidney neutrophil infiltration compared with infected WT kidneys. Kidney is the major target organ in disseminated candidiasis and is responsible for mouse mortality as shown previously [24,35,36,43,47,54,55]. The decreased recruitment of innate immune cells was associated with a decreased fungal burden at the sub-acute phase in the infected *Nos3*^*-/-*^ kidneys. Reduced inflammation due to a more balanced immunity by the absence of eNOS appears to be advantageous in controlling disseminated candidiasis.

The NanoString technique provides quantitative and sensitive detection of local changes in inflammatory gene expression in mouse kidneys [35,36]. iNOS and Il12a (IL12a) mRNAs were over-expressed 42- and 25-fold, respectively, compared with WT in *Nos3*^*-/-*^ mice kidneys at day 3. Although the fungicidal activity of RNS produced by iNOS may account for this protective activity, the mechanism by which the absence of eNOS enhances the induction of iNOS in infected kidneys remains to be determined.

Several genes in the IL-1 signaling pathway were differentially induced in infected *Nos3*^*-/-*^ compared with WT kidneys. The IL-1 pathway may also contribute to the more effective pro-inflammatory response in infected *Nos3*^*-/-*^ mouse kidneys infected with *C*. *albicans*. IL-1α & β induce a broad spectrum of immune inflammatory cell responses through the type I IL-1 receptor (IL-1R1) [56]. These include increased expression of TNF-alpha and iNOS mRNA together with signal transducer and activator of transcription-1 (STAT1) in infected *Nos3*^*-/-*^ kidneys, leading to pro-inflammatory reactions.

TLR-1, 2, 6 and 7 mRNAs were also up-regulated in *Nos3*^*-/-*^ kidneys. TLR induction stimulates signaling intracellular adaptors, such as MyD88 and TRIF, which in turn activate transcription factors like NF-κB and interferon regulatory factors (IRFs). The importance of TLR-mediated fungal recognition in host defense is supported by the susceptibility to disseminated candidiasis of MyD88-deficient mice [56].

We also found 25-fold increased IL-12a mRNA in *Nos3*^*-/-*^ kidneys compared with the WT. CD40 and STAT1 upregulation contribute to the Il-12 signaling pathway. IL-12 cytokine plays a role in both innate and adaptive immunity systems. Mouse model studies have shown that IL-12 induction contributes to the generation of Th1-type cytokine responses and protection against disseminated candidiasis [57].

Collectively, these data demonstrate that *Nos3*^*-/-*^ mice are more resistant to disseminated candidiasis and specifically associated with reduced renal pathology. Our finding that iNOS induced locally in kidneys of *Nos3*^*-/-*^ mice is associated with protection against disseminated candidiasis may have translational applications. *Nos3*^*-/-*^ mice exhibit attenuated pro-inflammatory responses to *Candida* infection in the kidney. Limiting eNOS activity may minimize destructive neutrophil infiltration into infected kidneys while locally up-regulating protective iNOS, increasing potentially protective Th9 cell recruitment, and systemically up-regulating IL-12 and IL-17. Therefore, a selective inhibitor of eNOS but not iNOS such as L-N^G^-monomethylarginine, which has been show to increase leukocyte adhesion and emigration [58], could be applied therapeutically to control disseminated candidiasis.

## Supporting information

S1 Fig Lack of kidney colonization at day 1 post-infection. Representative sections of mouse kidneys stained using GMS to detect *C*. *albicans* colonization (black staining) in cortex (A) and medulla (D) and H&E stained cortex sections (B) to show inflammatory reactions in *Nos3*^*-/-*^ kidneys compared with the WT at day 1 PI.
(PDF)

S1 File Minimal data set. Spread sheets used for calculating serum cytokine concentrations, kidney colony forming units, organ leukocyte flow cytometry data, and intracellular cytokine flow data.
(XLSX)
