# Supplementary material for: Endothelial nitric oxide synthase limits host immunity to control disseminated Candida albicans infections in mice
Source: PLoS One. 2019 Oct 31;14(10):e0223919. doi: 10.1371/journal.pone.0223919 (PMC6822743; doi:10.1371/journal.pone.0223919)
Supplement: S1 Fig — Representative sections of mouse kidneys stained using GMS to detect C. albicans colonization (black staining) in cortex (A) and medulla (D) and H&E stained cortex sections (B) to show inflammatory reactions in Nos3-/- kidneys compared with the WT at day 1 PI. (PDF) [file pone.0223919.s001.pdf]

Figure S1

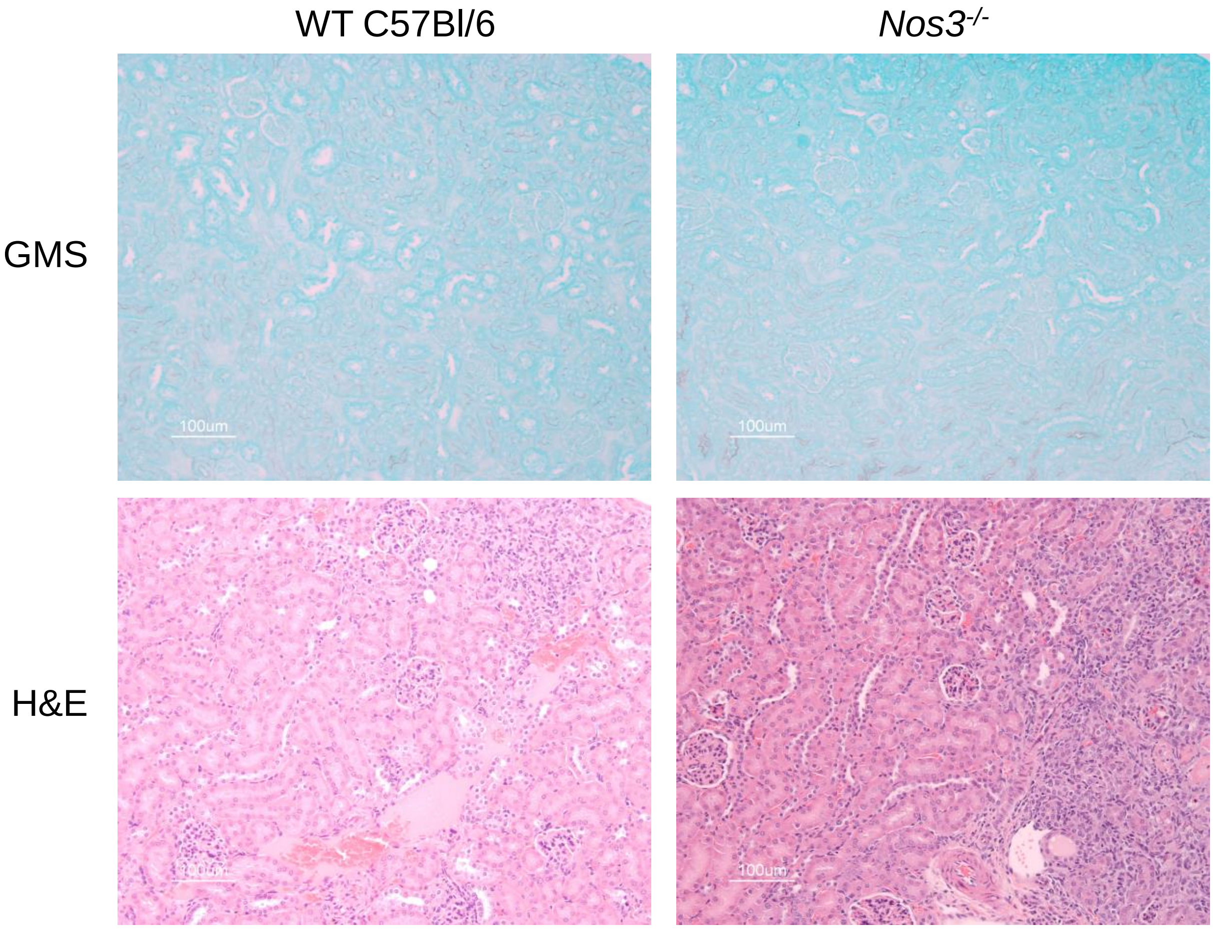

Figure S1. Representative sections of mouse kidney cortex from *Nos3*$^{-/-}$ or WT mice at day 1 PI were stained using GMS to detect *C*. *albicans* colonization (black staining) and H&E to show inflammatory reactions.
